# BASIC AND CLINICAL ASPECTS OF VASCULAR AGING

**DOI:** 10.1093/geroni/igae098.1473

**Published:** 2024-12-31

**Authors:** Cuntai Zhang

**Affiliations:** Tongji Hospital, Tongji Medical College, Huazhong University of Science and Technology, Wuhan, Hubei, China (People’s Republic)

## Abstract

Vascular aging (VA) is an important pathophysiological contributor to the aging process. In response to China’s aging population, a hierarchical “Standardized Vascular Aging Management Center (VMC)” network, with Tongji Hospital in Wuhan as the central hub, was established in 2023 to offer comprehensive vascular aging (VA) management nationwide. This initiative encompasses assessment, stratification, and intervention strategies for VA, a key factor in the aging of human organs and systems and a common denominator in the chronic diseases of aging. Our research delves into the cellular and molecular dynamics of VA, examining the effectiveness of pharmaceutical interventions against vascular stiffness and investigating cellular aging mechanisms such as metabolic changes, ferroptosis, mitochondrial dysfunction, and senescence-associated secretory phenotype (SASP), alongside key factors like SOX9, Nrf2, and FGF21. Furthermore, we are launching a national cohort study and conducting randomized trials on novel drugs to bridge clinical and basic research findings. This integrated approach aims to detect early VA signs, enabling timely interventions to mitigate chronic elderly diseases, foster healthy aging, and effectively tackle the implications of an aging demographic. By developing an integrated VA research and application platform that facilitates seamless translation between basic and clinical settings, we aim to identify early signs of VA and implement appropriate interventions to delay and manage this process. Ultimately, our efforts aim to reduce chronic disease in the elderly, promote healthy aging and more effectively address the challenges of an aging society.

